# Cardiovascular manifestations in idiopathic inflammatory myopathies

**DOI:** 10.1007/s10067-023-06599-4

**Published:** 2023-05-06

**Authors:** Meera Shah, Samuel Katsuyuki Shinjo, Jessica Day, Latika Gupta

**Affiliations:** 1 Department of Rheumatology, Indraprastha Apollo Hospital, New Delhi, Delhi 110076 India; 2 Division of Rheumatology, Faculdade de Medicina FMUSP, Universidade de São Paulo, São Paulo, SP Brazil; 3 Department of Rheumatology, Royal Melbourne Hospital, Parkville, VIC 3050 Australia; 4 Walter and Eliza Hall Institute of Medical Research, Parkville, VIC 3052 Australia; 5 Department of Medical Biology, University of Melbourne, Parkville, VIC 3052 Australia; 6 Department of Rheumatology, Royal Wolverhampton Hospitals NHS Trust, Wolverhampton, WV10 0QP UK; 7 Department of Rheumatology, City Hospital, Sandwell and West Birmingham Hospitals NHS Trust, Birmingham, UK; 8 Division of Musculoskeletal and Dermatological Sciences, Centre for Musculoskeletal Research, School of Biological Sciences, The University of Manchester, Manchester, UK

**Keywords:** Cardiac, Cardiovascular, Dermatomyositis, Heart, Myopathies, Myositis

## Abstract

Cardiovascular involvement in idiopathic inflammatory myopathies (IIM) is an understudied area which is gaining increasing recognition in recent times. Recent advances in imaging modalities and biomarkers have allowed the detection of subclinical cardiovascular manifestations in IIM. However, despite the availability of these tools, the diagnostic challenges and underestimated prevalence of cardiovascular involvement in these patients remain significant. Notably, cardiovascular involvement remains one of the leading causes of mortality in patients with IIM. In this narrative literature review, we outline the prevalence and characteristics of cardiovascular involvement in IIM. Additionally, we explore investigational modalities for early detection of cardiovascular involvement, as well as newer approaches in screening to facilitate timely management.

**Table:** 

**Key points** *• Cardiac involvement in IIM in majority cases is subclinical and a major cause of mortality.* *• Cardiac magnetic resonance imaging is sensitive for detection of subclinical cardiac involvement.*

**Supplementary Information:**

The online version contains supplementary material available at 10.1007/s10067-023-06599-4.

## Introduction

Idiopathic inflammatory myopathies (IIM) are heterogeneous, multisystem diseases with varied phenotype and presentation. Several subtypes are recognized, including dermatomyositis (DM), juvenile DM (JDM), clinically amyopathic DM (CADM), polymyositis (PM), inclusion body myositis (IBM), immune-mediated necrotizing myopathy (IMNM), anti-synthetase syndrome (ASS), and overlap myositis [1]. A diagnosis of IIM is associated with significant morbidity and an increased risk of mortality. Cancer, pulmonary involvement and cardiovascular disease are the most common causes of death worldwide [2–4]. Notably, a significant link between inflammation and cardiovascular involvement in rheumatic diseases has been described [5, 6]. Thus, patients with IIM are at an increased risk of cardiovascular involvement.

Despite advances in the management of patients with IIM, identification of cardiac involvement in these patients is often delayed. Limitations of clinical examination and traditional cardiac investigations have led to recognition of newer imaging modalities for the early detection of cardiac involvement in IIM.

This narrative review focuses on recent literature regarding cardiovascular involvement in patients with IIM, including its manifestation as a result of disease activity, cardiac inflammation and damage or comorbid cardiovascular risk factors. The authors discuss various investigational modalities for evaluating cardiac involvement with emphasis on the newer imaging modalities. This review aims to explore the undiscovered impact of cardiac involvement in patients with IIM, newer modalities for accurate identification and assessment, and the unmet need for standardized investigational protocol.

## Methods

**Fig. 1:**
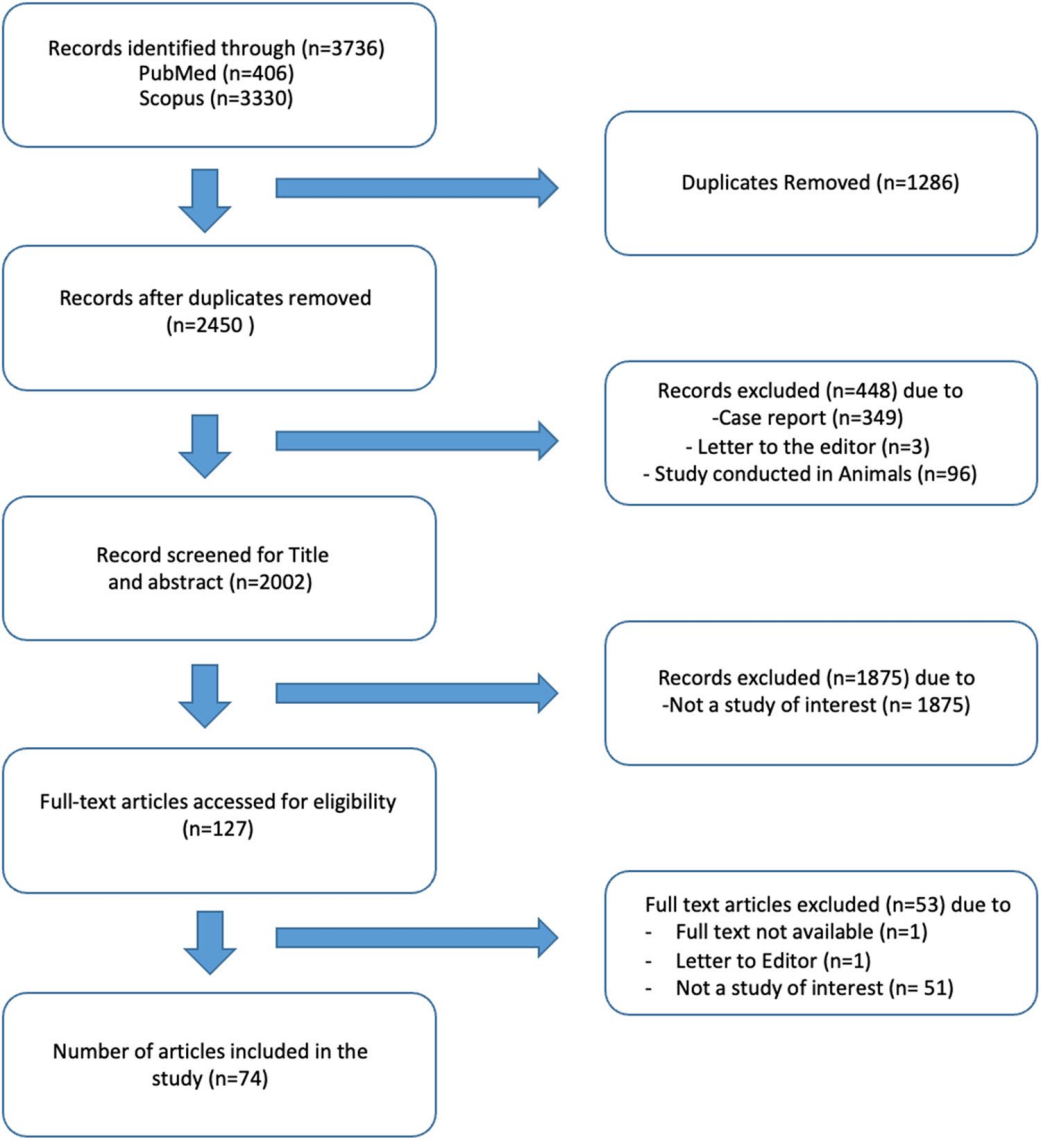
Workflow flowchart

The two largest medical databases, namely Scopus and PubMed were searched on the same day for relevant articles published between 2000 and 2022. The search terms used were [myositis OR dermatomyositis OR polymyositis OR inclusion body myositis OR idiopathic inflammatory myopathy OR autoimmune myopathy OR idiopathic inflammatory myopathies OR autoimmune myopathies] AND [cardiovascular risk OR heart failure OR cardiomyopathy OR cardiovascular disease OR myocarditis OR heart infarction OR heart disease OR heart arrhythmia OR coronary artery disease OR ischemic heart disease OR thromboembolism] as outlined in previously published methods protocols for narrative reviews [7]. Only data published in English were included. Case reports, case series with less than 10 cases, letter to editor, book chapters, and animal studies were excluded. Since most of the literature on myositis was covered in the above-mentioned databases, and Web of Science is rich in older literature and information in regional languages, these were considered beyond the scope of the authors’ current work. The detailed search strategy and results are outlined in Fig. 1. Additional studies were identified from references of the extracted articles and included where relevant.

### Epidemiology

Although first described in 1899 by Oppenheim, cardiac involvement in myositis was not a well-recognized manifestation until the 1970s [8, 9]. The understanding and definition of cardiac involvement in myositis have significantly evolved in recent years owing to the advent of more sensitive diagnostic techniques [10]. The prevalence of cardiac manifestations in IIM varies widely, ranging from 6 to 75% depending on the patient inclusion criteria, definition of cardiac involvement, and the imaging modalities used. Importantly, a substantial proportion of cardiac involvement in myositis is subclinical, with fewer than 10% being clinically manifest unless investigated [9]. The EuroMyositis registry, comprising 3067 cases across 11 countries, reported that cardiac involvement occurring due to the IIM disease process was most frequent in the connective tissue diseases-overlap myositis group and was least frequent in patients with IBM [11].

### Mortality and cardiovascular involvement (Table 1)

Several independent research groups worldwide have reported an increased risk of cardiovascular mortality in patients with IIM. For instance, a Swedish population-based nationwide cohort study by Dobloug et al. found that cardiac disease was one of the most common causes of mortality among IIM patients [2]. In a retrospective cohort study by Limaye et al., a standardized mortality ratio (SMR) of 1.75 was reported in IIM; the ratio was highest in the DM subtype at 2.40. The study also found that cardiovascular disease accounted for nearly one-third of deaths in the cohort of 364 individuals from South Australasia, followed by infection (22%) and malignancy (11%) [12]. Similarly, a large Korean population-based study of 3014 cases confirmed a high SMR and a high incidence of cardiovascular disease, even in young individuals with IIM (DM: 15.0, PM: 8.1) [14]. Dankó et al. conducted a survival analysis of 162 Hungarian patients and found that lung and cardiac complications were the most frequent causes of death. Death attributable to cardiovascular complications occurred late in the course of the disease (median 59 months). Cardiovascular manifestations were responsible for 8 out of 18 deaths (two cases of arrhythmia, three of heart failure, two of cardiac arrest, and one of myocardial infarction) and were identified as significant adverse prognostic factors [13].

**Table 1:** Mortality and cardiovascular involvement in patients with IIM

Author (year of publication)	Study group (*n*)	Diagnosis	Study design	Key observation	Country
Dobloug et al. [2] (2017)	716	IIM	Cohort study	IIM patients had a higher mortality rate compared to the general population. Malignancies, cardiac diseases and respiratory diseases were the main causes of death. Cardiac disease accounted for 28.1% of all deaths in IIM patients. The mortality rate was highest in the first year after the diagnosis of IIM	Sweden
Limaye et al. [12] (2012)	364	IIM	Retrospective Cohort study	A standardized mortality ratio (SMR) of 1.75 was reported in IIM, and it was highest in patients with DM (2.40). The major causes of death were cardiovascular disease (31%), infection (22%) and malignancy (11%). Risk factors for death included the absence of autoantibodies	Australia
Dankó et al. [13] (2004)	162	DM (*n* = 42) PM (*n* = 75) Juvenile (*n* = 9) Cancer associated myositis (*n* = 7) Overlap myositis (*n* = 29)	Cohort study	The most frequent causes of death were cardiac and pulmonary complications. Cardiovascular manifestations were responsible for 8 out of 18 deaths (two cases of arrhythmia, three of heart failure, two of cardiac arrest and one of myocardial infarction) and were significant prognostic factors for mortality. Deaths from cardiovascular disease occurred a median of 59 months post IIM diagnosis	Hungary
Jung et al. [14] (2020)	3014	DM (*n* = 1860) PM (*n* = 1154)	Population based study	Reported a very high SMR in young IIM patients (DM: 15.0, PM: 8.1). No influence of sex on the SMR was observed. Cardiovascular events were reported in 155 (5.1%) patients and 40.6% of patients with cardiovascular disease died	Korea

Collectively, these studies suggest cardiac complications are a common cause of mortality among IIM patients.

### Risk of cardiovascular disease

Accumulating evidence suggests an increased incidence of cardiovascular diseases such as acute coronary syndrome, heart failure, and arrhythmias in patients with IIM. Two meta-analyses (Xiong et al. and Ungprasert et al.) have examined the association between IIM and cardiovascular events in patients with PM and DM. The study by Xiong et al. included 25,433 patients with PM/DM and found a 2.3-fold increase in the risk of cardiovascular events within the first 5 years of IIM diagnosis. Moreover, PM/DM was identified as an independent risk factor for the development of cardiovascular events [15]. The study by Ungprasert et al., which comprised 13,201 patients, reported a 2.24-fold increased risk of cardiovascular disease in patients with IIM. Indeed, cardiovascular disease was identified as the leading cause of mortality in this cohort [16]. Prieto et al. conducted a study in the UK comprising 603 patients with IIM and reported a significantly greater rate of cardiovascular events in patients with IIM than in healthy controls, even after adjustment for traditional cardiovascular risk factors. This finding suggests that the increased risk could not be entirely attributed to traditional cardiovascular risk factors and was partly accounted for by the systemic inflammation present in these patients. The excess risk was most apparent in the first 5 years post IIM diagnosis and then appeared to decline [17]. These results are consistent with a case-control study which reported an increased risk of cardiovascular disease in patients with IIM, even in the absence of traditional risk factors [18]. Overall, the evidence suggests that patients with IIM have an increased risk of developing cardiovascular disease, particularly within the first 5 years of IIM diagnosis. This risk cannot be entirely attributed to traditional cardiovascular risk factors and may be partly due to systemic inflammation.

### Traditional cardiovascular risk factors (Table 2)

Diabetes mellitus, hypertension, and dyslipidemia are traditional risk factors for coronary heart disease. Several studies have investigated the prevalence of these risk factors in patients with IIM [12, 19, 21–27]. For instance, Diederichsen et al. observed a higher prevalence of hypertension (71% versus 42%) and diabetes mellitus (13% versus 0%) in patients with DM and PM compared to age- and sex-matched healthy controls. Patients with IIM also had significantly higher levels of serum triglycerides. Multivariate analysis revealed that smoking and age were associated with accelerated atherosclerosis in this cohort [20].

**Table 2:** Traditional cardiovascular risk factors in patients with IIM

Author (year of publication)	Study group (*n*)	Diagnosis	Study design	Key observation	Country
Wang et al. [19] (2014)	60	PM	Case control study	Duration of PM was less than 6 months and patients were treatment naive. 50% patients had reduced HDL levels and 47% patients had raised triglyceride levels. Serum CRP levels negatively correlated with HDL cholesterol. The inflammatory state in PM could be responsible for the metabolism of HDL cholesterol	China
Diederichsen et al. [20] (2015)	76	DM PM	Cross sectional observational study	Hypertension (71% versus. 42%) and diabetes mellitus (13% versus 0%) were more frequent in patients of DM and PM than in age- and sex-matched healthy controls. These patients also had significantly higher serum triglyceride levels. Presence of coronary artery atherosclerosis as evidenced by calcification on computed tomography scan was more prevalent in IIM patients, however, this was not associated with DM or PM	Denmark
Bae et al. [21] (2021)	95	DM (*n* = 55) PM (*n* = 30) IBM (*n* = 10)	Observational study	Worse HDL function was reported in patients with DM as opposed to patients with PM or IBM. Higher disease activity was associated with impaired antioxidant function of HDL	USA
Qin et al. [22] (2022)	-	IIM	Systematic review and Meta-analysis	Patients with IIM were 1.44, 1.67, and 1.48 times more likely to have hypertension, diabetes mellitus, and dyslipidemia respectively, when compared with non-IIM individuals	China
Pakhchanian et al. [23] (2021)	5578	DM	TriNetX registry	Of 5578 patients with DM, 66.82% of patients had hypertension as opposed to 25.05% of the general population. Similarly, the prevalence of ischemic heart disease (27.18% vs 7.3%) and diabetes mellitus (33.87% vs 12.14%) was higher in DM patients when compared to general population	
Oreska et al. [24] (2022)	39	DM-16 PM-7 IMNM-8 ASS-8	Cross sectional pilot study	Authors found no significant differences in the prevalence of traditional risk factors between patients with IIM and healthy controls	Czech Republic

This same study showed that the presence of coronary artery atherosclerosis, as evidenced by calcification on computed tomography scan, was more prevalent in IIM patients. However, this was associated with the presence of traditional cardiovascular risk factors rather than IIM-specific factors [20]. Conversely, Prieto et al. reported an increased risk of cardiovascular diseases in patients with IIM even in the absence of traditional cardiovascular risk factors [17]. A systematic review and meta-analysis by Qin et al. also reported that patients with IIM were 1.44, 1.67, and 1.48 times more likely to have hypertension, diabetes mellitus, and dyslipidemia respectively, when compared with non-IIM individuals [22]. In a case-control study by Wang et al., untreated patients with early PM (*n* = 60) had dyslipidemia characterized by high triglyceride and low high-density lipoprotein cholesterol levels, suggesting a high risk of atherosclerosis [19]. Similarly, a study by Pakhchanian et al. using the TriNetX database (5578 DM patients) found that 66.82% of patients had hypertension as opposed to 25.05% of the general population. The prevalence of ischemic heart disease (27.18% vs 7.3%) and diabetes mellitus (33.87% vs 12.14%) was also higher in DM patients when compared to the general population [23]. In contrast, a single-centre study (*n* = 39 patients) conducted by Oreska et al. found no significant differences in the prevalence of traditional risk factors between patients with IIM and healthy controls [24]. While mixed results have been reported, large cohort studies and meta-analyses all suggest an increased risk of cardiovascular risk factors in patients with IIM.

A possible mechanism for accelerated cardiovascular disease in IIM was explored in a study evaluating the antioxidant function of high-density lipoprotein (HDL) particles isolated from patient blood. The protective function of HDL in IIM patients was abnormal compared to healthy controls, with patients with DM and those with higher disease activity having particularly impaired HDL function [21]. Together, these studies underscore the need to determine the relative contribution of conventional risk factors vis-à-vis disease-specific inflammation-induced accelerated atherogenesis in the development of cardiovascular disease.

### Arrhythmias (Supplementary Table)

Various types of conduction system abnormalities have been described in patients with IIM, including supraventricular and ventricular tachycardia, fascicular blocks, bundle branch blocks, and atrioventricular (AV) blocks. In a large retrospective cohort study of 32,085 patients with DM or PM, Naaraayan et al. reported a significantly higher prevalence of atrial fibrillation, atrial flutter, supraventricular tachycardia, and unclassified arrhythmias in patients with IIMs < 70 years old compared with matched controls. The prevalence of arrhythmias was found to be higher in men than in women. Importantly, the diagnosis of arrythmias was associated with significant in-hospital mortality among patients with PM/DM [28]. A cross-sectional study of 112 IIM patients (78 DM and 34 PM) by Deveza et al. reported a higher frequency of arrhythmia in patients with PM when compared with DM. However, there was no significant difference in the occurrence of arrhythmias between IIM patients and the control group [29]. In a retrospective cohort study of 75 patients with IIM by Huang et al., PM and positive serum anti-mitochondrial antibodies (AMA) were strongly associated with ventricular arrhythmia in PM/DM patients with myocardial involvement [30].

Several mechanisms which may predispose to arrhythmias in patients with chronic inflammatory diseases such as IIM have been hypothesized, including infiltration of atria or ventricle by inflammatory cell infiltrate, autonomic dysfunction with resultant reduced heart rate variability and ‘acquired channelopathies’ due to release of cytokines or autoantibodies [31]. In a clinicopathological study of 16 autopsied patients with PM/DM conducted by Haupt et al., direct involvement of the conduction system by myositis and contraction-band necrosis was described [32].

### Thromboembolism (Supplementary Table)

Inflammation is known to play a role in promoting thrombosis by upregulating procoagulant pathways, downregulating natural anticoagulant factors and suppressing fibrinolysis [33]. An increased incidence of thrombosis in patients with chronic inflammatory diseases and autoimmune diseases is well described [34–38]. Patients with IIM are no exception, with several studies having reported a significantly increased risk of venous thromboembolism in patients with DM/PM [34, 39–42]. An enhanced in vitro thrombin generation profile in patients with DM/PM has been demonstrated, which may correlate to an increased risk of thromboembolic events [43]. Carruthers et al. reported an eight- and six-fold higher risk of developing venous thromboembolism in patients with DM and PM respectively. Notably, this risk was highest during the first year for patients with PM and during the initial 2 years after diagnosis in patients with DM, potentially attributable to uncontrolled inflammatory activity [44]. Similarly, in a retrospective analysis of 1144 Chinese patients with IIM, over half of IIM patients with thromboembolic events developed this complication in the 6 months before or after diagnosis of IIM. Increased myositis disease activity, malignancy, infection, and duration of steroid use were significant risk factors for thrombosis in this study [45]. A Swedish population-based cohort study (Antovic et al.), found that the risk of thromboembolic events was eight times higher in patients with IIM than in the general population, with the highest observed in patients with DM and a history of cancer. In line with other reports, the incidence rates were maximum in the first year post diagnosis and decreased thereafter [46]. Conversely, another retrospective cohort study found that age at diagnosis was the only independent risk factor for thrombotic events in patients with IIM. For each year of age, there was a 3.5% increased chance of experiencing a thrombotic event in patients with IIM. In their cohort of 253 patients with IIM, only 6% had anti-phospholipid antibodies, with no significant difference between the patients and comparators [47].

Pregnant women with autoimmune disease, including IIM, are also at an increased risk of thromboembolism. Indeed, Bleau et al. studied 43,523 pregnant women with autoimmune disease and observed that the risk of thromboembolism was particularly high for patients with DM comparted to other autoimmune diseases [48].

### Atherosclerotic cardiovascular diseases and acute coronary syndrome (Table 3)

Numerous studies have investigated the relationship between IIM and cardiovascular events. In a case-control analysis of 10,156 patients with DM conducted in the USA, researchers found that 20% of all DM hospitalizations were associated with an atherosclerotic cardiovascular diagnosis. Patients with DM and atherosclerotic heart disease were twice as likely to die during their hospital stay compared to DM patients who did not suffer from atherosclerotic heart disease (odds ratio (OR) 2.0, 95% confidence interval (CI) 1.7–2.5, *p* < 0.0001). Furthermore, among patients with both DM and cardiovascular disease, the odds ratio for death was 1.98 when compared to controls with only cardiovascular disease [49]. This suggests an additive effect of DM and cardiovascular disease on risk of death. Similarly, other studies have reported an increased risk of coronary heart disease in IIM patients when compared to controls [50, 56].

**Table 3:** Atherosclerotic cardiovascular disease and acute coronary syndrome in patients with IIM

Author (year of publication)	Study group (*n*)	Diagnosis	Study design	Key observation	Country
Linos et al. [49] (2013)	10156	DM	Case control analyses	20% of all DM hospitalizations were associated with atherosclerotic cardiovascular diagnosis. Heart failure (12% of DM hospitalizations) was the most common followed by myocardial infarction (4.4% of DM hospitalizations). When compared to DM patients without atherosclerotic heart disease, patients with associated atherosclerotic heart disease were twice more likely to die during hospital stay (OR = 2.0 95% CI 1.7–2.5, *p* < 0.0001). In those patients with both dermatomyositis and cardiovascular disease the odds ratio for death was 1.98 when compared to controls with only cardiovascular disease	USA
Weng et al. [50] (2019)	1145	DM (*n* = 640) PM (*n* = 505)	Retrospective population-based cohort	The authors reported adjusted hazard ratios (aHR) of 2.21 (95% CI 1.64, 2.99) in DM and 3.73 (95% CI 2.83, 4.90) in PM for coronary heart disease. There was a 2- and 3-fold increase in the risks of incident CHD in patients with DM and PM respectively when compared to their respective comparison groups	Taiwan
Leclair et al. [51] (2019)	655	DM (*n* = 218) Other IIM (*n* = 437)	Population based cohort study	This population-based cohort study reported that patients with IIM experienced their first ACS episode earlier than general population comparators identified from national registries and matched for follow up period [median (interquartile range) 2.4 (1.0–4.6) vs 3.5 (1.8–6.0) years)]. The hazard ratio was 2.4 for ACS in IIM patients compared to general population, with an increased risk in the first year of diagnosis	Sweden
Rai et al. [52] (2016)	774	DM (*n* = 350) PM (*n* = 4242)	Matched cohort analyses	This matched cohort analysis reported a nearly 4-fold increased risk of myocardial infarction in patients with PM and a 3-fold increased risk in DM patients when compared with controls	Canada
Lin et al. [53] (2015)	2029	DM/PM	Population based retrospective cohort study	The authors reported a 1.74 times greater incidence of ACS in a DM/PM cohort of 2029 patients. This incidence was greater in men than in woman. Patients with hypertension and end stage renal disease had significantly greater risk of developing ACS (aHR 2.48, 95% CI 1.37–4.52; aHR 4.86, 95% CI 1.41–16.88, respectively). The authors postulated that the occurrence of ACS in these patients may be attributed to mechanisms other than traditional cardiovascular risk factors as a higher risk of ACS (HR 1.75) was observed in DM/PM patients without comorbidities when compared with non-DM/PM patients	Taiwan
Lai et al. [54] (2013)	907	DM	Prospective cohort study	The authors reported an adjusted hazard ratio (3.37) of acute myocardial infarction close to the crude hazard ratio (3.96) in 907 patients of DM, implying that DM plays an independent role in increased cardiovascular events	Taiwan
Tisseverasinghe et al. [55] (2009)	607	DM/PM	Nested case control analyses	An association between the incidence of myocardial infarction and stroke and the presence of hypertension and lipid abnormalities in patients with DM/PM was observed. Immunomodulators such as methotrexate, azathioprine, antimalarial agents, cyclophosphamide) were negatively associated with these arterial events	Canada

Patients with IIM are at a significantly increased risk of experiencing an acute coronary syndrome (ACS) when compared to the general population as evidenced by several studies [51–54]. A study by Lin et al. reported a greater incidence of ACS in men than in women, and in those with hypertension and end stage renal disease. However, the authors also noticed an higher risk of ACS [Hazard ratio (HR) 1.75] in DM/PM patients without comorbidities when compared to the non-DM/PM cohort, leading them to postulate that mechanisms other than traditional cardiovascular risk factors may contribute to the increased ACS risk in these patients [53]. In line with previously mentioned study, Lai et al. reported an adjusted HR (3.37) for acute myocardial infarction close to the crude hazard ratio (3.96) in 907 patients of DM, implying that DM plays an independent role in increased cardiovascular events [54]. Finally, a nested case-control analysis by Tisseverasinghe et al. reported an association between the incidence of myocardial infarction and stroke, hypertension and lipid abnormalities. On the other hand immunomodulators such as methotrexate, azathioprine, antimalarial agents, cyclophosphamide were negatively associated with these arterial events [55].

Thus, factors besides traditional cardiovascular risk factors might contribute to the increased risk of cardiovascular disorders in patients with IIM; a finding which needs to be explored further.

### Heart failure (Supplementary Table)

Heart failure is the most frequently reported clinically apparent cardiovascular manifestation in patients with IIM [9, 57]. In a retrospective cohort study in Taiwan, patients with PM/DM showed a significantly increased risk of heart failure as compared to those without PM/DM (HR 2.06, 95%CI 1.36–3.12). The risk of heart failure was found to be highest within first 10 years following diagnosis of PM/DM [58]. In a cross-sectional multicenter study of 108 patients with IIM and with myocardial involvement, 62% of patients presented with heart failure, 56.5% with clinically significant arrhythmia, and 18.5% of patients presented with both. Anti-mitochondrial antibodies and pulmonary hypertension were significant associated factors [59]. In a single center retrospective study of 32 Chinese patients with IIM and heart failure, 13 of the 17 recorded deaths were attributed to cardiogenic shock [60].

### Myocarditis (Supplementary Table)

Diagnosing myocarditis in patients with inflammatory myopathies poses a challenge due to its subclinical nature in the majority of cases [9]. The prevalence of myocarditis in IIM has been reported to be 38% on cardiac pathology [61]. Conversely, Dieval et al. reported that the prevalence of clinically apparent was 3.4% in patients with ASSD. Myocarditis was the initial presenting feature in 42% of the ASSD patients with myocarditis in this study. Myocarditis was reported to be associated with active disease but not with any particular antibody specificity [62]. Liu et al. reported a shorter disease duration, more manifestations of heart failure and symptoms of IIMs and increased frequency of AMA-M2 antibody positivity in patients of IIM with myocarditis [63].

### Autoantibodies

Emerging data indicate an association between cardiovascular disease and certain myositis-specific antibodies, myositis-associated antibodies and anti-mitochondrial antibodies [64–66]. In a longitudinal cohort study comprising 619 adult and 371 juvenile patients with IIM, 31 adult patients were diagnosed with cardiomyopathy. While only 5% of adult patients in this cohort were AMA-positive, the presence of this antibody was associated with the development of cardiomyopathy and persistent muscle weakness. Notably, this association was not seen in the pediatric population [67]. Additionally, a retrospective case review reported cardiac involvement in five of seven patients with positive anti-mitochondrial antibodies. The authors noted that cardiac function in patients with cardiomyopathy or myocarditis could improve with immunosuppression [68]. In another study of 63 patients with IIM (DM, PM, CADM), those who were anti-MDA5 (melanoma differentiation-associated protein 5) positive (*N* = 21) showed significantly lower T waves amplitude on the electrocardiogram (ECG) than anti-MDA-5 negative patients. Nonetheless, there were no differences in the contractile function of the heart between the two groups. These low amplitudes were restored during the remission phase, suggesting a possible role for aberrant immune responses associated with active anti-MDA5+ DM [69].

### Juvenile dermatomyositis (Table 4)

JDM, although rare, is the most common childhood inflammatory myopathy. There are several similarities between the clinical features of JDM and those of adult DM [78].

**Table 4:** Cardiovascular manifestations in Juvenile Dermatomyositis

Author (year of publication)	Study group (*n*)	Diagnosis	Study design	Key observation	Country
Coyle et al. [70] (2009)	17	JDM (*n* = 16) Juvenile Polymyositis [JPM (*n* = 1)]	Cohort study	Authors reported a high frequency of metabolic abnormalities and metabolic syndrome in patients with JDM. 71% of patients had a blood pressure greater then 75^th^ percentile, and 47% patients had BMI > 85^th^ percentile. Hypertriglyceridemia was present in 47.1% of patients. Metabolic abnormalities appeared to be linked to disease activity	USA
Silverberg et al. [71] (2018)	1407	JDM		Hypertension was the most common comorbidity in children with JDM. Even in the absence of traditional cardiovascular risk factors, children with JDM had an increased risk of cardiovascular comorbidities when compared to non-JDM patients. Thus, traditional cardiovascular risk factors may not completely account for the increased rates of cardiovascular comorbidities in JDM	USA
Kozu e al. [72] (2013)	25	JDM		Kozu et al studied the lipid profile of 25 patients with JDM. Compared to healthy controls, patients with JDM had significantly higher triglyceride levels and lower median high density lipoprotein levels. The authors reported a positive correlation between dyslipidemia and disease activity	Brazil
Witczak et al. [73] (2022)	57	JDM	Cross-sectional study	In this study, 18% of JDM patients had cardiac involvement, which was mostly subclinical. Patients with cardiac involvement showed higher disease activity 1 year after diagnosis and an unfavorable lipid profile	Norway
Witczak et al. [74] (2022)	59	JDM	Cross-sectional study	This study demonstrated higher body fat percentage and lower appendicular lean mass index in patients with active JDM when compared to children with inactive JDM. Central fat distribution was linked to cardiometabolic alterations, notably left ventricular dysfunction	Norway
Barth et al. [75] (2019)	58	JDM		The authors did not find any significant differences in the ECG, heart rate variability, and systolic or diastolic function between patients with low and normal NCD. However, they reported an association between lung involvement and low NCD. A plausible explanation for the lack of association between NCD and cardiac dysfunction could be the small sample size of the study, making the study underpowered to demonstrate this association	Norway
Schwartz et al. [76] (2013)	59	JDM	Cross-sectional study	In this cross-sectional study of 59 JDM patients examined at a median of 16.8 years after disease onset, both systolic and diastolic dysfunction were reported to be linked to longer disease duration and high disease activity score of skin but not muscle at 1 year. Cumulative prednisolone dose was also a predictor of diastolic dysfunction. Thus, sustained early skin activity could predict cardiac dysfunction in long term	Norway
Barth et al. [77] (2016)	55	JDM		Barth et al demonstrated lower heart rate variability (HRV) in patients with active JDM than inactive JDM. This reduced HRV was found to be associated with elevated inflammatory markers, reduced myocardial function and active disease	Norway

Cardiovascular risk factors and metabolic syndrome appear prevalent among patients with JDM. For instance, several studies have observed a high frequency of hypertension [70, 71], dyslipidemia [70–73] and elevated body fat percentage [70, 74] in JDM patients versus controls. In several studies, metabolic abnormalities were found to be correlated with JDM disease activity [70, 72]. Importantly, increased rates of cardiovascular involvement have been observed even in the absence of traditional cardiovascular risk factors, suggesting that such risk factors may not completely account for the increased rates of cardiovascular disease in JDM [71].

Potential cardiac manifestations in JDM include pericarditis, systolic or diastolic dysfunction and electrocardiographic abnormalities [75, 76, 79]. In a cross-sectional study of 57 patients with JDM, 18% of the patients had cardiac involvement- defined as left ventricular systolic or diastolic dysfunction on echocardiography, which was mostly subclinical. Patients with cardiac involvement showed higher disease activity 1 year after diagnosis and an unfavorable lipid profile [73]. In a cross-sectional study of 59 patients with JDM examined at a median of 16.8 years after disease onset, both systolic and diastolic dysfunction were reported to be linked to longer disease duration and high disease activity score of skin but not muscle at 1 year. The cumulative prednisolone dose was also a predictor of diastolic dysfunction. Thus, sustained early skin activity could predict cardiac dysfunction in the long term [76].

A low heart rate variability (HRV) is a strong predictor of cardiac arrest [80]. Barth et al. demonstrated a lower HRV in patients with active JDM than in those with inactive JDM, indicating the presence of autonomic nervous system dysregulation in JDM. This reduced HRV was found to be associated with elevated inflammatory markers, reduced myocardial function, and active disease [77].

Abnormal nailfold capillary density (NCD) is an indicator of systemic vasculopathy and thus may have utility in the evaluation of JDM. Barth et al. did not find any significant differences in the electrocardiography, heart rate variability, and systolic or diastolic function between patients with low and normal NCD. However, these authors reported an association between lung involvement and low NCD. A plausible explanation for the lack of association between NCD and cardiac dysfunction could be the small sample size of the study, making the study underpowered to demonstrate this association [75].

### Investigations (Table 5)

Given that cardiovascular involvement is one of the most common causes of increased morbidity and mortality in patients with IIM, screening of patients for cardiac involvement is of paramount importance. As most patients have subclinical cardiac involvement, the need for highly sensitive and noninvasive testing methods is gaining importance.

**Table 5:** Investigational findings in patients with IIM (key studies)

Author (year of publication)	Study group (*n*)	Diagnosis	Study design	Key observation	Country
Electrocardiogram (ECG)					
Deveza et al. [29] (2014)	112	DM (*n* = 78) PM (*n* = 34)	Cross-sectional study	One-third of patients had ECG abnormalities namely conduction disorders, chamber enlargement and rhythm disturbances. The rhythm disturbances included ventricular extrasystole, atrial fibrillation, first-degree AV block, supraventricular tachycardia, supraventricular extrasystoles. All these abnormalities were more frequent in PM than in DM (50% vs. 24.4%, *p* = 0.008). The study, however, did not find any significant difference in ECG abnormalities between patients and controls, except for a higher prevalence of left ventricular hypertrophy in the former	Brazil
Triplett et al. [81] (2020)	109	IMNM	Retrospective study	In patients with IMNM, an abnormal ECG was documented in 55 out of 86 patients. Of the various abnormalities, prolongation of corrected QT interval (QTc) was the most frequent	USA
Cox et al. [82] (2010)	51	Sporadic IBM	Cross-sectional study	Patients with IBM did not have increased risk of cardiac involvement compared to general population as evidenced by the similar frequencies of ECG abnormalities between the two groups	Netherlands
Wang et al. [83] (2014)	51	DM		Patients with DM had no clinically evident cardiovascular disease assessed using Doppler ECG. Authors reported a statistically significant association between LVDD and duration of the disease, pointing towards subclinical cardiac involvement with disease advancement	China
Nuclear Cardiac Imaging					
Diederichsen et al. [84] (2016)	76	DM (*n* = 24) PM (*n* = 52)	Cross-sectional study	Age, disease duration, presence of myositis specific or associated autoantibodies and high cardiac 99m Tc-PYP uptake were found to be associated with LVDD. This association of LVDD with increased cardiac 99m Tc-PYP uptake indicated myocardial inflammation as a primary cause of cardiac involvement in IIM patients	Denmark
Echocardiography					
Plazak et al. [85] (2011)	15	DM/PM	Cross-sectional study	This study of patients with autoimmune disease included 15 DM/PM patients. Pathologic valvular leaflet thickening and/or pericardial thickening was found in 46.7% of patients with DM/PM. Pericardial effusion was also reported in 66.7% of DM/PM patients. Patients with DM/PM had dilated right ventricle with elevated right ventricular systolic pressure	Poland
Zhong et al. [86] (2017)	60	DM/PM	Cross-sectional study	In a study of 60 PM/DM patients with preserved LVEF, speckle tracking echocardiography demonstrated subtle systolic dysfunction. The severity of cardiac involvement was related to the systemic disease burden	China
Guerra et al. [87] (2017)	28	DM/PM	Case control study	The authors reported a 4.9-fold higher risk of subclinical ventricular systolic dysfunction in IIM patients compared to healthy controls using speckle tracking echocardiography. The basal and mid-segments of the anterior, anterior-septal, and lateral wall of the left ventricle were most frequently involved. The presence of cardiac involvement was not associated with disease duration or disease activity	Italy
Liu et al. [88] (2022)	46	DM/PM	Observational study	Using speckle tracking echocardiography, it was noted that in patients with PM and DM, the myocardium at the base was more severely affected than at the apex. This pattern of basal weakness accurately differentiated myocardial involvement of DM and PM from acute viral myocarditis	China
Cardiac magnetic resonance					
Khoo et al. [89] (2019)	19	DM (*n* = 4) PM (*n* = 4) IBM (*n* = 2) IMNM (*n* = 2) Myositis not otherwise specified (*n* = 4) Overlap myositis (*n* = 2) DM/SSc overlap (*n* = 1)	Cohort study	Despite being asymptomatic for cardiac disease, almost 50% of patients with IIM had apparent cardiac involvement on CMR in the form of late gadolinium enhancement (LGE). Except for one patient, all patients showed varying degrees of cardiac fibrosis	Australia
Sun et al. [90] (2021)	51	DM (*n* = 19) PM (*n* = 20) NM (*n* = 12)	Observational study	This study demonstrated more severe LGE lesions in PM patients when compared to patients with DM. Patient with necrotizing myositis (NM) did not demonstrate late gadolinium enhancement	China
Kersten et al. [91] (2021)	47	DM (*n* = 10) PM (*n* = 31) Other sub-forms of IIM (*n* = 6)	Cohort study	Patients with IIM had lower myocardial deformation parameters (indicative of impaired myocardial function) when compared to healthy volunteers using cardiac magnetic resonance imaging. In this study there was no significant difference in the LVEF between patients and healthy volunteers, suggesting that change in myocardial deformation may precede changes in LVEF	Germany
Rosenbohm et al. [92] (2020)	20	IBM	Case series of 20 patients	In this case series of 20 patients with sporadic IBM, CMR demonstrated decreased left and right ventricular stroke volumes and an increased early myocardial enhancement when compared to controls. These changes were attributed to the hypertensive heart disease present in these patients. There was no statistical difference in LGE between patients and controls	Germany
Cardiac enzymes					
Lillekar et al. [93] (2018)	123	DM (*n* = 39) PM (*n* = 34) ASS (*n* = 37) IMNM (*n* = 8) IIM-CTD overlap disease (*n* = 5)	Cross sectional study	Cardiac troponin I (cTnI) levels were higher in patients with cardiac involvement, irrespective of disease activity. cTnI had the highest specificity (95%) and positive predictive value (62%) for detecting cardiac involvement, however, it lacked sensitivity. Cardiac troponin T (cTnT) correlated with the patient and evaluator global assessment and the quality of life as assessed by Health Assessment Questionnaire (HAQ) more strongly than cTnI and creatinine kinase (CK)	UK and Denmark
Sun et al. [90] (2021)	51	DM (*n* = 19) PM (*n* = 20) IMNM (*n* = 12)	Observational study	In a study of 51 IIM patients (19 patients with DM, 20 patients with PM and 12 patients with IMNM), patients with IMNM had very high serum markers of cardiac damage (CK-MB and cTnT) as compared to patients with PM and DM. The study showed that serum CK-MB and cTnT did not accurately reflect myocardial involvement in IIM, however, NT-pro BNP correlated positively with CMR finding of LGE.	China

#### Electrocardiogram (ECG)

Electrocardiographic changes are common in patients with IIM [94], with abnormalities detected in approximately 32.5–72% of patients [9]. In a study by Deveza et al., one-third of patients with DM and PM had electrocardiographic abnormalities including conduction disorders, chamber enlargement, and rhythm disturbances. The various rhythm disturbances observed were ventricular extrasystoles, atrial fibrillation, first-degree AV block, supraventricular tachycardia, and supraventricular extrasystoles. These abnormalities were more frequent in PM than in DM (50% versus 24.4%, *p* = 0.008]. However, the study did not find any significant difference in electrocardiographic abnormalities between patients and control groups, except for a higher prevalence of left ventricular hypertrophy in the former [29]. In a study of IMNM, 55 out of 86 patients had an abnormal electrocardiography. Of the various abnormalities, prolongation of the corrected QT interval (QTc) was the most frequent [81].

Conversely, patients with sporadic inclusion body myositis do not appear to have increased risk of cardiac involvement compared to the general population, as evidenced by the similar frequencies of ECG abnormalities between the two groups [82].

#### Echocardiography

Echocardiographic abnormalities are reported in 14–65% of patients with IIM [3]. Left ventricular diastolic dysfunction (LVDD) is the most common echocardiographic abnormality [61, 81, 84, 95, 96]. Several studies have demonstrated that IIM patients have a greater prevalence of LVDD compared to the general population [83, 84, 95]. A study by Wang et al. reported a statistically significant association between LVDD and disease duration, pointing towards increased subclinical cardiac involvement with disease advancement [83]. Diederichsen et al. reported that age, disease duration, presence of myositis-specific or myositis-associated autoantibodies, and high cardiac 99m Tc-PYP uptake are associated with LVDD. This association between LVDD and increased cardiac 99m Tc-PYP uptake indicated myocardial inflammation as a primary cause of cardiac involvement in patients with IIM [84].

In a study of patients with generalized autoimmune disease (comprising 60 patients with scleroderma, 60 patients with systemic lupus erythematosus, and 15 patients with DM/PM) pathologic valvular leaflet thickening and/or pericardial thickening on echocardiography was found in 46.7% of patients with DM/PM. Pericardial effusion was also reported in 66.7% of the patients with DM/PM. All 15 patients with DM/PM had dilated right ventricle with elevated right ventricular systolic pressure [85].

Left ventricular systolic function has long been assessed using left ventricular ejection fraction (LVEF). However, LVEF has limited sensitivity in detecting early subclinical cardiac involvement, as it typically does not worsen until cardiac function is severely compromised. Speckle tracking echocardiography is an emerging modality for detection of subclinical cardiac involvement by directly assessing myocardial wall deformation [97]. Echocardiographic assessment using the long axis strain and not LVEF for evaluating systolic involvement in patients with IIM has been recommended [94].

In a study of 60 patients with PM/DM with preserved LVEF, speckle-tracking echocardiography demonstrated subtle systolic dysfunction. The severity of cardiac involvement was related to the systemic disease burden [86]. Guerra et al. reported a 4.9-fold higher risk of subclinical ventricular systolic dysfunction in patients with IIM than to healthy controls using speckle tracking echocardiography. The basal and mid-segments of the anterior, anteroseptal, and lateral walls of the left ventricle were the most frequently involved. However, in their study, the cardiac involvement was not associated with disease duration or disease activity [87]. In a study by Liu et al. using speckle tracking echocardiography, it was noted that in patients with PM and DM, the myocardium at the base was more severely affected than at the apex. This pattern of basal weakness was successful in accurately differentiating myocardial involvement of DM and PM from acute viral myocarditis [88].

#### Nuclear cardiac imaging

Nuclear cardiac imaging can be used to detect myocarditis and myocardial damage from past ischaemic insults. A retrospective study by Okada et al. examined the usefulness of ^123^I-BMIPP, which reflects fatty acid metabolism, and ^201^TlCl (201 thallium chloride), which reflects myocardial perfusion, scintigraphy for detecting myocarditis in 26 PM or DM patients. Half of the patients demonstrated ^123^I-BMIPP/ ^201^TICI mismatch, and these patients had significantly lower LVEF. They suggested ^123^I-BMIPP and ^201^TICI nuclide scintigraphy could be contemplated for the evaluation of myocarditis in patients with IIM [98].

#### Cardiac magnetic resonance

Cardiac magnetic resonance (CMR) imaging is considered the most reliable noninvasive modality for the detection of myocarditis and myocardial fibrosis in patients with IIM [57]. Khoo et al. reported that almost 50% of patients with IIM had apparent cardiac involvement on CMR imaging, as evidenced by late gadolinium enhancement (LGE), despite being asymptomatic for cardiac disease. Except for one patient, all participants demonstrated varying degrees of fibrosis in this study [89]. Huber et al. demonstrated that CMR T1 mapping parameters in myocardial and skeletal muscles can be used to differentiate between acute viral myocarditis and IIM related myocarditis [99]. An observational study by Sun et al. demonstrated more severe LGE lesions in patients with PM than in patients with DM. Conversely, patients with IMNM did not demonstrate late gadolinium enhancement [90]. Kersten et al. reported lower myocardial deformation parameters in patients with IIM than in healthy volunteers. Notably, there was no significant difference in the LVEF between patients and healthy volunteers, suggesting that changes in myocardial deformation may precede changes in LVEF [91].

Cardiac magnetic resonance (CMR) imaging has shown promise in the early detection of cardiac involvement in patients with IIM. A case series of 53 patients with PM, DM or non-specific myositis reported myocardial LGE patterns that were consistent with inflammation. Despite a normal LVEF, 54.5% of the patients had positive LGE. There was no significant correlation between the clinical symptoms and CMR results [92]. Several studies have demonstrated the superiority of multiparametric CMR approaches to conventional methods (ECG and echocardiography) in early detection of cardiac involvement, even in patients with negative LGE [100–102]. Based on their study evaluating CMR in patients of IIM at first diagnosis and after a year of receiving standard treatment, Xu et al. suggested that multiparametric CMR could help monitor response to effective treatment [103]. In a case series of 20 patients with sporadic IBM, CMR demonstrated decreased left and right ventricular stroke volumes and increased early myocardial enhancement compared to controls. However, these changes were attributed to hypertensive heart disease in these patients. Of note, there was no statistical difference in the LGE between patients and controls [104].

#### Cardiac enzymes

Cardiac troponins are highly sensitive markers of cardiac involvement, particularly infarction. A retrospective study by Aggarwal et al. reported elevated cardiac troponin T (cTnT), but not cardiac troponin I (cTnI), in patients with IIM. cTnT levels correlated with total creatine kinase (CK) levels in this study [105]. Of note, cTnT can arise from the skeletal muscles of patients with IIM, which may explain why this enzyme correlates with muscle disease activity [93, 106]. Conversely, in a study by Lilleker et al., cTnI levels were higher in patients with cardiac involvement, irrespective of IIM disease activity. cTnI had the highest specificity (95%) and positive predictive value (62%) for the detection of cardiac involvement, although this test lacked sensitivity. cTnT correlated more strongly with the patient and evaluator global assessment and the quality of life as assessed by the Health Assessment Questionnaire (HAQ) than cTnI and CK [93].

In a study of 51 patients with IIM (19 patients with DM, 20 patients with PM, 12 patients with IMNM) patients with IMNM had significantly higher cTnT and CK, but lower N-terminal prohormone of brain natriuretic peptide (NT-proBNP) levels and no positive LGE on CMR when compared to patients with PM and DM, again suggesting that serum CK-MB and cTnT do not accurately reflect myocardial involvement in IIM. NT-pro BNP correlated positively with CMR finding of LGE [90].

Although several diagnostic methods are available for investigating cardiac involvement in patients with IIM, traditional methods such as ECG and echocardiography are not sensitive to detect subclinical and early changes. CMR has proven to be sensitive for the detection of subclinical cardiac involvement, the most frequently encountered cardiac abnormality. Data on nuclear cardiac imaging for detection of myocarditis in patients with IIM is very limited, and needs to be studied further.

## Conclusion

Cardiovascular risk factors are prevalent in individuals diagnosed with IIM. Although clinically apparent cardiac involvement is uncommon in patients with IIM, cardiovascular involvement is a major contributor to morbidity and mortality. It remains unclear whether the excess cardiovascular morbidity and mortality seen in IIM is a result of increased prevalence of cardiovascular risk factors, chronic systemic inflammation or subclinical cardiac inflammation. Further research may help understand the relative contribution of these mechanisms.

Of concern, subclinical cardiac involvement may not be identified using conventional investigations such as electrocardiography or echocardiography. Advances in highly sensitive imaging platforms such as CMR, coupled with the increasing availability of these modalities has made it easier to detect subclinical cardiac involvement; however, larger studies are needed to assess the feasibility of employing such imaging modalities in the routine screening of patients with IIM. This scoping review identifies a pressing need to develop guidelines for screening cardiac involvement and cardiovascular risk factors in patients with IIM.

### Supplementary information

ESM 1. (DOCX 18 kb)
