# Supplementary material for: Cardiovascular manifestations in idiopathic inflammatory myopathies
Source: Clin Rheumatol. 2023 May 6;42(10):2557–75. doi: 10.1007/s10067-023-06599-4 (PMC10497702; doi:10.1007/s10067-023-06599-4)
Supplement: Supplementary file 1 — (DOCX 18 kb) [file 10067_2023_6599_MOESM1_ESM.docx]

**Supplementary Table:**

**Title: “Cardiovascular manifestations in Idiopathic Inflammatory Myopathies”**

**Journal Name:** Clinical Rheumatology

**Authors:**

*Meera Shah^1^*

*Samuel Katsuyuki Shinjo^2^*

*Jessica Day^3, 4, 5^*

*Latika Gupta ^6, 7, 8^*

**Affiliations:**

*^1.^* Department of Rheumatology, Indraprastha Apollo Hospital, New Delhi, Delhi-110076, India. ORCiD: 0009-0005-3308-7231.

*^2.^* Division of Rheumatology, Faculdade de Medicina FMUSP, Universidade de São Paulo, São Paulo, SP, Brazil. ORCiD: 0000-0002-3682-4517

^3.^ Department of Rheumatology, Royal Melbourne Hospital, Parkville, VIC 3050, Australia. ORCiD: 0000-0001-8528-4361.

^4.^ Walter and Eliza Hall Institute of Medical Research, Parkville, VIC 3052 Australia. ORCiD: 0000-0001-8528-4361.

^5.^ Department of Medical Biology, University of Melbourne, Parkville, VIC 3052 Australia. ORCiD: 0000-0001-8528-4361.

^6.^ Department of Rheumatology, Royal Wolverhampton Hospitals NHS Trust, Wolverhampton, UK. ORCiD: 0000-0003-2753-2990

^7.^ Department of Rheumatology, City Hospital, Sandwell and West Birmingham Hospitals NHS Trust, Birmingham, UK. ORCiD: 0000-0003-2753-2990

^8.^ Division of Musculoskeletal and Dermatological Sciences, Centre for Musculoskeletal Research, School of Biological Sciences, The University of Manchester, Manchestet, UK. ORCiD: 0000-0003-2753-2990

**Corresponding author’s details:**

**Dr. Latika Gupta**

Email- [drlatikagupta@gmail.com](mailto:drlatikagupta@gmail.com)

Supplementary Table:

| Author  Year of publication | | Study group (n) | Diagnosis | Study Design | Key observation | Country |
| --- | --- | --- | --- | --- | --- | --- |
| Arrhythmias in patients with IIM | | | | | | |
| Naaraayan et al [28]  2021 | | 32,085 | PM  DM | Retrospective Cohort study | Higher prevalence of atrial fibrillation (17.3%), atrial flutter (2.7%), supraventricular tachycardia (2.4%) and unclassified arrythmias (3.7%) compared to controls. A higher prevalence of arrhythmias was observed in patients <70 years of age. In addition, the prevalence of arrhythmias was higher in men than in women. The authors also found increased in-hospital mortality in patients with PM/DM with arrhythmias compared to patients without a diagnosis of arrhythmia. | USA |
| Deveza et al [29]  2016 | | 112 | DM (n=78)  PM (n=34) | Single-centre, cross-sectional study | A higher frequency of arrhythmias was reported in patients with PM when compared to DM. Atrial fibrillation, first degree atrioventricular block and supraventricular extrasystoles were found only in patients with PM. However when compared to control group, there was no significant difference in the occurrence of conduction disturbances in IIM patients. | Brazil |
| Huang et al [30]  2021 | | 75 | DM (n=31)  PM (n=44) | Retrospective Cohort Study | PM and positive serum anti-mitochondrial were strongly associated with ventricular arrhythmia in patients with PM/DM and myocardial involvement. Ventricular arrhythmia and heart failure were recognized as independent prognostic factor in PM/DM patients with myocardial involvement. | China |
| Thrombotic manifestations in patients with IIM | | | | | | |
| Carruthers et al [44]  2016 | 752 | | DM (n=355)  PM (n=443)  Both (n=46) | Matched Cohort Analyses | Patients with DM and PM had 8- and 6-fold higher risk of developing venous thromboembolism (deep venous thrombosis and pulmonary embolism) respectively. This risk was highest during the first year for PM patients and initial 2 years after diagnosis in patients with DM. This increased risk of thrombosis in initial years of disease diagnosis may be attributable to uncontrolled inflammatory activity. | Canada |
| Chen et al [45]  2022 | | 1144 | IIM | Retrospective Study | 24 patients (2.1%) had thromboembolic events (arterial or venous). 13 out of 24 patients (54.2%) had thromboembolic events 6 months before or after the diagnosis of IIM. Multivariable regression model showed higher disease activity, malignancy, longer duration of glucocorticoid use and malignancy as significant risk factors for thrombosis. | China |
| Antovic et al [46]  2018 | | 440 | DM (n=154)  Other IIM (n=286) | Population based cohort study | The risk of thromboembolic events was 8 times more in patients with IIM compared to general population. The risk was highest in patients with DM and those with a history of cancer. The incidence rates were maximum in the first year of diagnosis and decreased thereafter. | Sweden |
| Notarnicola et al [47]  2021 | | 51 cases | DM (n=20)  PM (n=22)  IBM (n=9)  ASS (n=10) | Retrospective cohort study | Following multivariate logistic regression analysis, age at diagnosis was the only independent risk for thrombotic events in IIM patients. For each year of age, there was a 3.5% increased chance of experiencing a thrombotic event in a patient with IIM. In this cohort only 6% of patients had anti-phospholipid antibodies, with no significant difference between the patients and comparators. | Sweden |
| Heart failure in patients with IIM | | | | | | |
| Lin et al [58]  2022 | | Unmatched cohort (n=2025)  Propensity score matched cohort (n= 1997) | DM & PM | Retrospective Cohort Study | Patients with PM/DM showed a significantly increased risk of heart failure when compared to non PM/DM population (HR: 2.06, 95% CI: 1.36–3.12). This risk appeared to highest within the initial years (upto 10 years) of diagnosis of PM/DM. This study indicated an increased risk of hospitalization for heart failure in patients with DM/PM as duration of disease increased. | Taiwan |
| Zhang et al [59]  2021 | | 1946 | DM & CADM (n=1562)  PM (n=385) | Multicentre cross-sectional study | Of 108 IIM patients with myocardial involvement, 62% presented with heart failure, 56.5% with clinically significant arrhythmia and 18.5% of patients presented with both. Positivity for anti-signal recognition particle (SRP) (14.8 vs 6.8%, P = 0.026), anti-SSB (8.8 vs 4.0%, P = 0.021), anti-mitochondrial antibody (AMA) (16.4 vs 2.8%, P < 0.001), and AMA-M2 (24.6 vs 5.1%, P < 0.001) was significantly higher in the myocardial involvement group compared to the control group. | China |
| Liu et al [60]  2020 | | 32 | DM (n=11)  PM (n=14)  Overlap syndrome (n=7) | Single centre retrospective study | This study included 32 patients with IIM with heart failure. Systolic dysfunction and restrictive diastolic function were the classical features on echocardiography. 13 out of 17 deaths were cardiogenic in nature. | China |
| Myocarditis in patients with IIM | | | | | | |
| Dieval et al [62]  2015 | | 12 | Anti-synthetase syndrome | Retrospective study | Reported a myocarditis prevalence of 3.4% in patients with anti-synthetase syndrome (ASS). Of ASS patients with myocarditis, it was the presenting feature in 42% of cases. Myocarditis was reported to be always associated with active disease. The prevalence of myocarditis was not linked to any particular autoantibody | France |
| Liu et al [63]  2020 | | 62 | DM (n=28)  PM (n=34) | Single centre observational retrospective cohort study | Reported a shorter disease duration, more manifestations of heart failure and symptoms of IIMs and increased frequency of AMA-M2 antibody positivity in patients of IIM with myocarditis. | China |
